# Reduced anticipation of negative emotional events in alexithymia

**DOI:** 10.1038/srep27664

**Published:** 2016-06-09

**Authors:** Francesca Starita, Elisabetta Làdavas, Giuseppe di Pellegrino

**Affiliations:** 1 Department of Psychology, University of Bologna, Italy; 2 CSRNC, Center for Studies and Research in Cognitive Neuroscience, University of Bologna, Italy

## Abstract

Alexithymia is characterized by difficulties in different domains of emotion processing, especially in relation to negative emotions. Nevertheless, its causal mechanisms remain elusive. Reduced anticipation of negative emotional events might be one such mechanism because it enables the individual to prepare to respond effectively to coming events. To test this, changes in skin conductance response (SCR) were recorded during classical fear conditioning in sixty participants with high (HA), medium (MA) and low (LA) levels of alexithymia. Two coloured squares were presented, one was reinforced with a mild electrical stimulation (CS+) while the other was never reinforced (CS−). Critically, despite all groups showing higher SCR to CS+ compared to CS−, SCR to CS+ was lower and extinguished earlier in HA compared to MA and LA. These differences appeared to be attributable neither to differences in the intensity of stimulation received, nor to SCR to the stimulation itself. Groups showed comparable SCR to CS− as well. Therefore, HA exhibited decreased anticipation of the occurrence of a negative emotional event. Disruption of this mechanism may then compromise effective emotion recognition, emotional response and response regulation, which characterise HA, and represent a unifying causal mechanism underlying the difficulties in emotion processing of this group.

Alexithymia is a personality trait characterised by difficulties in identifying and describing feelings and discriminating between feelings and bodily sensations of emotional arousal^1^,^2^. Although considered a subclinical phenomenon, higher prevalence of high levels of alexithymia than the general population have been found in a number of conditions, including anxiety, depression, eating disorders and substance abuse, leading to the hypothesis that HA might represent a risk factor for developing such pathologies^3^. In addition, the incidence of high alexithymia appears higher also in autism compared to the general population^4^, and there is consistent evidence showing that the emotional difficulties observed in autism may in fact be due to co-occurring alexithymia^5^,^6^,^7^. Given this, it seems crucial to understand the role played by high alexithymia in emotion processing.

Research is producing a large body of literature on the differences exhibited by individuals with high levels of alexithymia (HA) as compared to individuals with low levels of alexithymia (LA), especially in relation to negative emotions. For example, HA are less accurate in recognizing emotional faces presented for a brief period of time^8^,^9^, rate the expression of fearful faces as less intense^10^, and fail to remap fear on their own somatosensory system^11^. Moreover, decreased activation of the amygdala has been reported during the processing of emotional stimuli in HA^12^,^13^, which seems to be specific to negative stimuli, such as sad faces, fearful bodies or observation of pain in others^14^,^15^,^16^,^17^. They also show impairments in emotional response regulation^18^ appearing less able to recur to reappraisal as a strategy to regulate emotions^19^. Furthermore, HA exhibit decreased empathic concern leading to more inclination towards utilitarian decisions in moral dilemmas^20^.

Although relating to distinct aspects of emotion processing, the difficulties of HA could be partly caused by a unifying underlying mechanism. Reduced anticipation of emotional events might be hypothesised to be one such mechanism. In fact, the anticipation of emotional events is a crucial adaptive mechanism, which enables the individual to prepare to respond effectively to the coming events. Through learning processes, individuals become able to attribute an emotional value to previously neutral cues and, as a consequence, anticipate an emotional event from cues in the environment, which have now become predictors of its occurrence^21^,^22^. By anticipating the coming emotional event, individuals not only can form a cognitive representation of the coming event per se but also of its consequences. The former then enables faster recognition and response to the emotional event, the latter optimal emotional response regulation and decision making^23^. Therefore, despite being a low level process, learning to anticipate emotional events might be crucial for effective emotion processing. Disruption of this process has been observed in psychopathologies related to emotion processing, such as depression, anxiety or psychopathy^24^,^25^,^26^. Similarly, this could be true also for HA.

Classical fear conditioning has been extensively used to study the process of learning to anticipate the occurrence of negative emotional events^27^. There, a neutral stimulus is paired with an aversive unconditioned stimulus (UCS), which elicits innate emotional responses, named unconditioned response (UR). After repeated pairing of the two stimuli, the individual learns to anticipate the occurrence of UCS at the presentation of the neutral stimulus, which becomes a conditioned stimulus (CS). In the end, the sole presentation of CS elicits an anticipatory response in preparation to the occurrence of UCS, called conditioned response (CR). Both UR and CR are marked by physiological changes in autonomic nervous system activity and increased skin conductance response (SCR) represents one of them^28^,^29^. Higher SCR in response to CS signals increased expectations regarding the occurrence of UCS following the presentation of CS indicating that the association between CS and UCS has been learnt^30^. Additionally, changes in subjective affective experience accompany the physiological changes and higher arousal and lower pleasantness are generally reported by participants at the presentation of CS compared to a neutral stimulus^31^.

Therefore, classical fear conditioning could be used to test whether HA present a reduced response in anticipating the occurrence of negative emotional events. To this end, sixty participants with HA, LA and medium levels of alexithymia (LA), as measured by the 20-item Toronto Alexithymia Scale (TAS-20)^32^, completed a classical fear conditioning task with partial reinforcement^33^,^34^. On each trial, one of two coloured squares was presented on a computer screen for 6 seconds followed by an inter trial interval of 12 seconds. The task included 40 trials (20 for each stimulus) divided in three blocks: habituation, acquisition and extinction. During habituation (4 trials) none of the stimuli was reinforced to ensure there were no baseline differences in response to the stimuli. During acquisition (16 trials) one stimulus was reinforced with a mild electric stimulation (UCS) on 80% of trials (CS+) while the other was never reinforced (CS−). During extinction (20 trials) no stimulation was administered. Changes in SCR were recorded continuously during the experiment as a somatic indicator of the degree of anticipation of the UCS. In addition, to assess subjective experience, participants reported the level of anxiety and fear experienced at the presentation of each CS during the experiment. Finally, because anxiety is known to affect SCR in classical conditioning^25^, levels of anxiety were measured with the State-Trait Anxiety Inventory^35^ and correlations between levels of anxiety and differential SCR response were explored to exclude an effect of anxiety on results. HA were hypothesised to show decreased anticipation of the electrical stimulation following the presentation of CS+, hence exhibit lower SCR to CS+ compared to MA and LA. No differences between MA and LA were expected.

## Results

### UCS intensity and peak and mean SCR to UCS

Univariate ANOVAs were used to evaluate differences in UCS intensity and mean and peak SCR to the UCS. Results showed no significant differences among the three groups in either UCS intensity (*M*_*LA*_ = 4.49 μS, *SD*_*LA*_ = 2.86 μS; *M*_*MA*_ = 4.01 μS, *SD*_*MA*_ = 2.52 μS; *M*_*HA*_ = 3.33 μS, *SD*_*HA*_ = 2.16 μS; *F*(2, 57) = 1.06, *p* = 0.353, partial η^2^ = 0.04), peak SCR in response to UCS (*M*_*LA*_ = 1.24 μS, *SD*_*LA*_ = 0.56 μS; *M*_*MA*_ = 1.26 μS, *SD*_*MA*_ = 0.56 μS; *M*_*HA*_ = 1.14 μS, *SD*_*HA*_ = 0.46 μS; *F*(2, 57) = 0.31, *p* = *0*.736, partial η^2^ = 0.01) or mean SCR in response to UCS (*M*_*LA*_ = 1.03 μS, *SD*_*LA*_ = 0.48 μS; *M*_*MA*_ = 1.08 μS, *SD*_*MA*_ = 0.46 μS; *M*_*HA*_ = 0.91 μS, *SD*_*HA*_ = 0.38 μS; *F*(2, 57) = 0.80, *p* = 0.456; partial η^2^ = 0.03). On average, the intensity of the stimulation received by participants as well as the physiological response to it did not differ significantly among groups.

### SCR during habituation

**Figure 1:**
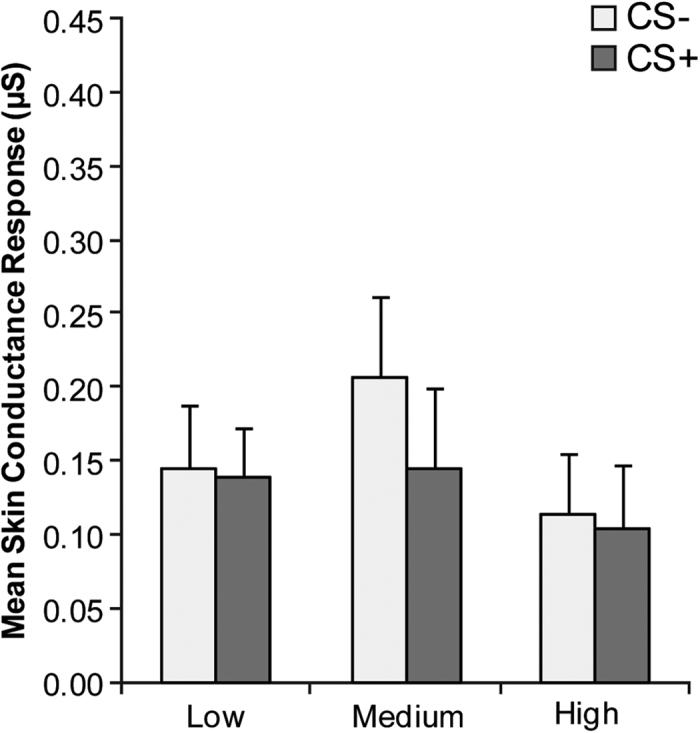
Habituation. Mean skin conductance response (SCR) to the two conditioned stimuli (CS−, CS+) during habituation as a function of alexithymia group. Error bars represent standard errors.

A 3 × 2 RM ANOVA was carried out to analyse habituation with group as between-subject variable (low, medium, high) and stimulus type as within-subject variable (CS−, CS+). Analysis on SCR showed no significant main effect of group (*F*(2, 57) = 0.71, *p* = 0.494, partial η^*2*^ = 0.02), stimulus (*F*(1, 57) = 0.84, *p* = 0.361, partial η^2^ = 0.01) or interaction (*F*(2, 57) = 0.38, *p* = 0.681, partial η^2^ = 0.01), confirming that at baseline there were neither within group nor between group differences in response to the two conditioned stimuli (Fig. 1).

### SCR after habituation

**Figure 2:**
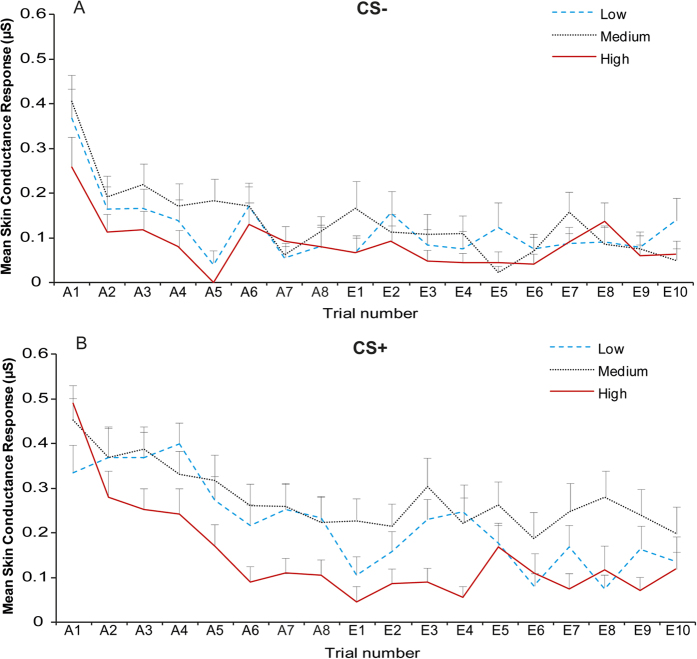
Skin conductance response during acquisition and extinction. Trial by trial mean skin conductance response (SCR) to the two conditioned stimuli (panel A: CS−; panel B: CS+) as a function of alexithymia group. Error bars represent standard errors.

Figure 2 shows the mean SCR of the groups to CS− (panel A) and CS+ (panel B) for each trial of acquisition and extinction. A 3 × 2 × 3 RM ANOVA was carried out to analyse SCR in the phases following habituation with group as between-subject variable (low, medium, high) and stimulus (CS−, CS+) and phase (acquisition, early extinction and late extinction) as within-subject variables. Analysis on SCR showed significant stimulus by phase by group interaction (*F*(4,114) = 3.64, *p* = 0.008, partial η^2^ = 0.11). This interaction was further explored conducting separate ANOVAs for acquisition and extinction.

### SCR during acquisition

**Figure 3:**
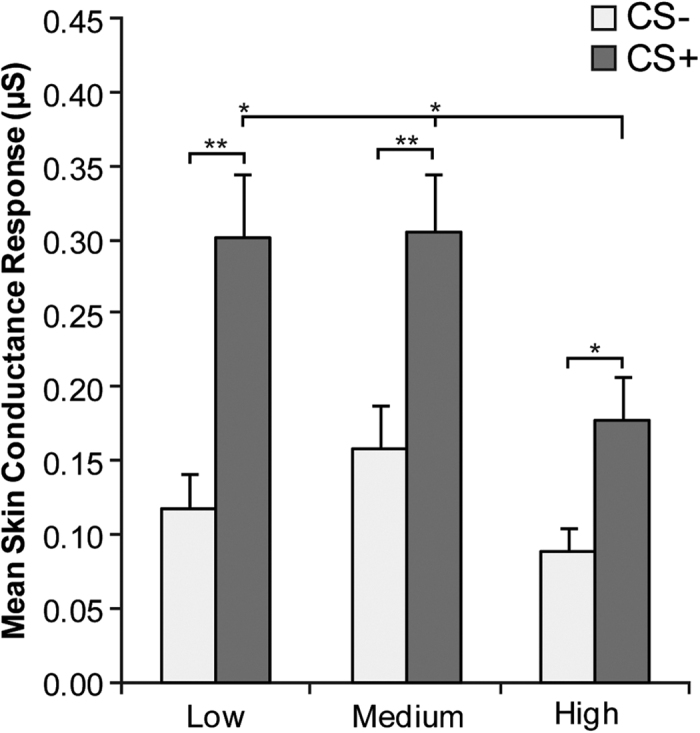
Acquisition. Mean skin conductance response (SCR) to the two conditioned stimuli (CS−, CS+) during acquisition as a function of alexithymia group. Error bars represent standard errors. Significant differences are indicated as follows: *p < 0.05; **p < 0.001.

During acquisition, a significant stimulus by group interaction (*F*(2, 57) = 3.26, *p* = 0.046, partial η^2^ = 0.10) was found, indicating that groups differed in SCR to the two conditioned stimuli during acquisition. Newman-Keuls test showed that despite all groups showing significant difference in response to CS+ as compared to CS− (*M*_*LACS*−_ = 0.12 μS, *SD*_*LACS*−_ = 0.12 μS; *M*_*LACS*+_ = 0.30 μS, *SD*_*LACS*+_ = 0.19 μS; *p* < 0.001; *M*_*MACS*−_ = 0.16 μS, *SD*_*MACS*−_ = 0.13 μS; *M*_*MACS*+_ = 0.31 μS, *SD*_*MACS*+_ = 0.17 μS; *p* < 0.001; *M*_*HACS*−_ = 0.09 μS, *SD*_*HACS*−_ = 0.07 μS; M_HACS+_ = 0.18 μS, *SD*_*HACS*+_ = 0.13 μS; *p* = 0.007), there was a significant difference between groups in response to CS+. Specifically, HA had significantly lower SCR compared to LA (*p* = 0.007) and MA (*p* = 0.015). No difference was found in SCR to CS+ between LA and MA or in response to CS− among any of the groups (all *p* > 0.262; Fig. 3). Therefore, all groups showed differential SCR to CS+ compared to CS−. However, SCR to CS+ exhibited by HA was significantly lower than SCR exhibited by the other two groups. On the contrary, responses to CS− were comparable among groups.

The main effect of group (*F*(2, 57) = 3.26, *p* = 0.046, partial η^2^ = 0.10) and stimulus (*F*(1, 57) = 84.74, *p* < 0.001, partial η^2^ = 0.60) were also significant. However, these were secondary to the interaction described above.

Given that difference in SCR between CS+ and CS− may be influenced by the levels of anxiety, correlations between these two variables were explored to exclude a significant contribution of anxiety to the results. Neither trait nor state anxiety correlated significantly with difference in SCR to the two conditioned stimuli (all *p* > 0.292).

### SCR during extinction

Extinction was divided in two blocks, early and late to investigate the role of time in the extinction of the conditioned response. A 3 × 2 × 2 RM ANOVA was carried out to analyse extinction with group as between subject variable and stimulus type and time (early, late) as within-subject variables. Nevertheless, analysis showed that time did not play a significant role in extinction. Neither a significant main effect nor interaction of time with the other factors was found (all *p* > 0.183).

**Figure 4:**
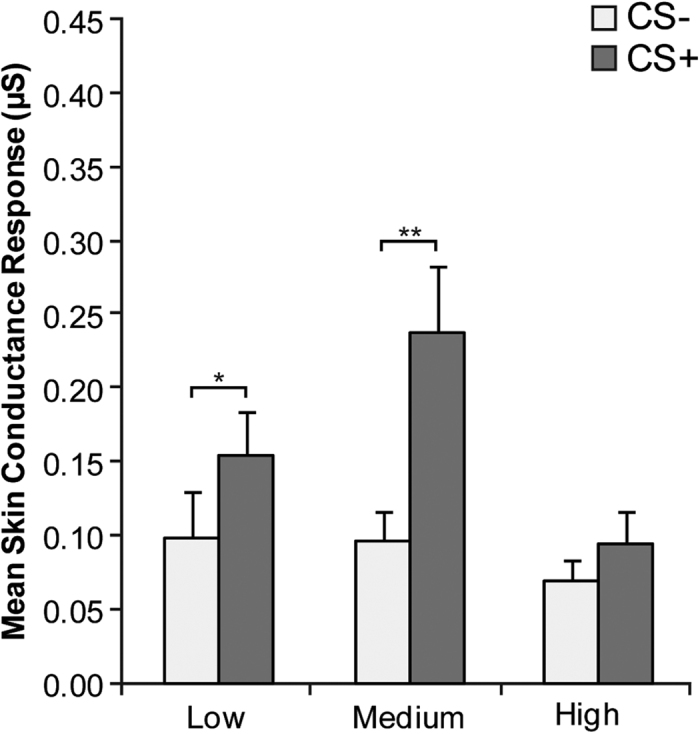
Extinction. Since the factor time did not interact significantly with the other factors, the figure shows mean skin conductance response (SCR) to the two conditioned stimuli (CS−, CS+) during extinction as a function of alexithymia group collapsing early and late extinction blocks. Error bars represent standard errors. Significant differences are indicated as follows: *p < 0.05; **p < 0.001.

On the contrary, there was a significant stimulus by group interaction (*F*(2, 57) = 5.53, *p* = 0.007, partial η^*2*^ = 0.16). Newman-Keuls test showed that only LA and MA maintained a significantly higher SCR to CS+ compared to CS− (*M*_*LACS*−_ = 0.10 μS, *SD*_*LACS*−_ = 0.15 μS; *M*_*LACS*+_ = 0.15 μS, *SD*_*LACS*+_ = 0.14 μS; *p* = 0.037; *M*_*MACS*−_ = 0.10 μS, *SD*_*MACS*−_ = 0.09 μS; *M*_*MACS*+_ = 0.24 μS, *SD*_*MACS*+_ = 0.20 μS; *p* < 0.001; Fig. 4).

A main effect of stimulus was present (*F*(1, 57) = 24.30, *p* < 0.001, partial η^*2*^ = 0.30), although secondary to the interaction described above. Instead, the main effect of group resulted non significant (*F*(2, 57) = 2.63; *p* = 0.081, partial η^*2*^ = 0.08).

Also in this phase neither trait nor state anxiety correlated significantly with the difference in SCR between CS+ and CS− (all *p* > 0.616).

### Subjective reports of anxiety and fear

3 × 2 RM ANOVAs were conducted on subjective reports of fear and anxiety experienced at the presentation of the conditioned stimuli for each phase of conditioning with group as between-subject variable (low, medium, high) and stimulus type as within-subject variable (CS−, CS+). Both the subjective report on anxiety and fear showed a main effect of stimulus (respectively: *F*(1, 57) = 170.41, *p* < 0.001, partial η^*2*^ = 0.75; *F*(1, 57) = 133.34, *p* < 0.001, partial η^*2*^ = 0.70). The reported anxiety to CS+ (*M*_*CS*+_ = 59.84%, *SD*_*CS*+_ = 21.39%) was higher than to CS− (*M*_*CS*−_ = 23.56%, *SD*_*CS*−_ = 18.29%; *p* < 0.001) as well as the reported fear to CS+ (*M*_*CS*+_ = 46.13%, *SD*_*CS*+_ = 23.05%) was higher than to CS− (*M*_*CS*−_ = 13.41%, *SD*_*CS*−_ = 13.22%; *p* < 0.001). In contrast, no significant main effect of group or interaction was found either for anxiety or fear.

## Discussion

This study investigated whether HA presented reduced anticipation of negative emotional events. To this end, changes in SCR were recorded during classical fear conditioning to assess differences among LA, MA and HA in anticipating the occurrence of a negative emotional event by learning patterns of association between CS+ and UCS.

All Participants correctly associated CS+ and UCS, suggesting that they explicitly identified the stimulus that anticipated the negative emotional event. In addition, groups did not differ in the intensity of UCS received, SCR to it and emotional experience reported in response to presentation of CS+. On the contrary, results showed significant differences among HA and MA and LA in SCR during acquisition that exacerbated during extinction. Specifically, during acquisition all three groups learned the anticipatory value of CS+ in predicting UCS, as indicated by higher SCR to CS+ compared to CS−. However, the degree of physiological response elicited by the anticipation of UCS in HA was lower compared to MA and LA, as shown by significantly lower SCR to CS+. This reduced response intensified during extinction, when the differential SCR to CS+ extinguished in HA while it was maintained in MA and LA. This suggested that the response elicited by the anticipation of UCS disappeared as soon as the predictive value of CS+ was no more reinforced by the administration of UCS. Crucially, this result did not appear to be dependent solely on the reduced SCR to CS+ during acquisition. These differences between the groups were attributable neither to differences in the intensity of UCS, because all groups received comparable intensities of stimulation, nor to reactivity to UCS itself, because groups did not differ in mean or peak SCR amplitude to UCS. In addition, groups did not differ in their SCR during habituation, acquisition or extinction to CS−, indicating comparable physiological response to neutral stimuli as well. Therefore, although HA seem to learn to differentiate a neutral from a conditioned stimulus, they appear less responsive in anticipating the negative consequences of a conditioned stimulus compared to LA and MA. This becomes particularly evident once the conditioned stimulus ceased to be reinforced revealing a difficulty in maintaining the association learned over time. As soon as the conditioned stimulus was no more reinforced by the aversive stimulus, the emotional value that HA had learnt to attribute to the conditioned stimulus disappeared.

Physiological changes in the anticipation of negative emotional events have been proposed to be a crucial component of emotional experience^36^ and they have the adaptive function of guiding attention towards the source of the events preparing the organism to effectively identify, respond and regulate the response to such event^22^,^28^. Therefore, the anticipation of emotional events might be crucial for effective emotion processing. Results suggest that HA are less able to anticipate the coming emotional event and possibly its consequences, which would be crucial to allow rapid identification, response and regulation of the response to such event. This difference may represent a shared underlying mechanism contributing to the difficulties of this group in emotion processing, which are particularly evident in ambiguous contexts, such as the recognition of emotional stimuli during limited time constraints, decision making in moral dilemmas and emotional response regulation^8^,^9^,^18^,^20^.

Anticipating the emotional future seems to involve more complex mechanisms than just learning about the contiguity between CS+ and UCS^30^. In fact, the individual is required to learn the causal relationship between the CS+ and UCS. At each learning trial, UCS acts as teaching signal strengthening the response to CS+. The strength of this teaching signal is modulated by predictions regarding the occurrence of UCS following the presentation of CS+^37^. A brain circuit seems to be responsible for such process involving the periacqueductal gray, relaying the UCS teaching signal to the amygdala through indirect pathways via the thalamus, which then project to the medial prefrontal cortex (mPFC) and anterior cingulate cortex (ACC). Once in the amygdala, the UCS teaching signal then modulates plasticity at CS+ input synapses strengthening the response to CS+^37^. The amygdala then sends an output to the regions that regulate activity in the autonomic nervous system, to generate changes in SCR^38^,^39^,^40^. Indeed, previous research has shown decreased activation of mPFC, ACC and amygdala during processing of negative emotional stimuli in HA^16^,^41^. Similarly, this circuit might be less active also during the anticipation of negative emotional events.

The lower physiological response in anticipation to UCS in HA was not reflected in lower subjective reports of fear and anxiety. HA reported comparable levels of anxiety and fear experienced at the presentation of CS+ to LA and MA. These data might seem to contrast with an influential account of alexithymia, which describes alexithymia as the emotional equivalent of blindsight^42^. According to this frame, alexithymia would be characterised by an intact physiological response and a deficit in emotion concept representation^43^. Nevertheless, the literature concerning this aspect has reported inconsistent results. Studies found both comparable^44^,^45^,^46^ and decreased^47^,^48^,^49^,^50^,^51^ physiological response to emotional stimuli together with no difference^48^, increased^50^,^52^,^53^ or decreased^46^ subjective reports of emotional experience. To reconcile these contrasting findings, the literature has hypothesised that alexithymia might be characterised by a decoupling between the subjective experience and physiological response to emotional stimuli^52^ and the present data would support this decoupling. However, the direction of this decoupling remains a matter for future investigation. In addition, it has been argued that processes generating the physiological response in fear conditioning interact with but are distinct from those that give rise to conscious feelings of fear and anxiety^22^,^28^. In fact, while the amygdala is a crucial structure in generating SCR to CS+, cortical areas seem to be involved in attributing meaning to interoceptive inputs to construct the experience of an emotion^54^. Speaking more broadly, the present data suggest that the processes giving rise to the explicit emotional experience might be partly dissociated from those giving rise to the physiological response to emotional stimuli. This dissociation has been observed in a number of other conditions. For example, patients with lesions to the amygdala have shown diminished^55^ or absent^56^ SCR to an aversively conditioned stimulus, despite intact unconditioned response and awareness about the association between conditioned and unconditioned stimulus. On the contrary, patients with split brain^57^, hemispatial neglect^58^ and affective blindsight^59^,^60^,^61^ have shown intact physiological response in the absence of awareness for emotional stimuli. Nevertheless, although physiological responses and awareness for emotion can be separated, somatic and interoceptive information regarding one’s own body is generally incorporated with semantic and contextual knowledge to generate an integrated representation of affective state^62^,^63^ and this might be the case for LA and MA. However, in HA the physiological and cognitive aspect of emotional experience may remain decoupled possibly contributing to their difficulties. Despite comparable cognitive aspects of emotional experience, lower physiological response in anticipation of emotional events alone might not be sufficient to prepare HA to effectively respond to emotional events.

To conclude, the present study shows that HA are less able to anticipate the occurrence of negative emotional events compared to LA and MA. This indicates a disruption in HA in learning to attribute an emotional value to previously neutral stimuli and use them as cues to predict the emotional future. The ability to predict the emotional future has the adaptive function of guiding attention towards the source of the emotional event preparing the organism to effectively respond to it^21^,^22^,^28^. Anticipating the coming emotional event, individuals not only can form a cognitive representation of the coming event but also of its consequences. Therefore, disruption of this process may lead to difficulties in effective recognition, response and response regulation to emotional events, which characterise HA and may represent a unifying low level mechanism, which may underlie part of the difficulties in emotion processing of HA. As this represents the first evidence of disruption in emotional learning in HA, further research will be needed to clarify which aspect of emotional learning might be affected in HA and in what way this can impact higher level emotion processing.

## Methods

### Participants

Three-hundred university students completed the 20-item Toronto Alexithymia Scale (TAS-20)^32^. Depending on the score, students were classified as LA (TAS-20 ≤ 36), MA (36 < TAS-20 < 61) or HA (TAS-20 ≥ 61)^64^. Individuals from the three groups were randomly contacted and asked to participate in the study. Due to the high co-occurrence of alexithymia and depression^65^, participants completed the Beck Depression Inventory^66^, and were excluded in case their score was higher than the moderate/severe depression cut-off (i.e. 19; *n* = 5). Sixty-two university students with no history of neurological or psychiatric disorders completed the study. After the experimental task, explicit awareness of the contingency between CS and UCS was assessed. Two participants were removed from analysis due to failure in reporting the correct association between stimuli. The final sample included in the analysis consisted of 60 participants (22 males, 38 females; age *M* = 24.03, *SD* = 2.38 years old) divided in three groups: 20 LA participants (13 females; TAS-20 *M* = 30.42, *SD* = 3.79; age *M* = 24.67, *SD* = 2.83 years old); 20 MA participants (12 females; TAS-20 *M* = 46.10, *SD* = 6.18; age *M* = 24.06, *SD* = 1.80 years old); 20 HA participants (13 females; TAS-20 *M* = 63.63, *SD* = 2.39; age *M* = 23.35, *SD* = 2.32). A priori targets for sample size and data collection stopping rule were based on sample and effect sizes reported in the literature on classical fear conditioning (sample size around 17–19 participants per group as indicated by a recent meta-analysis^67^).

Because anxiety is known to affect SCR in classical conditioning^25^, levels of anxiety were measured with the State-Trait Anxiety Inventory^35^. Levels of anxiety in the three groups differed significantly both for state (*F*(2, 57) = 5.86, *p* = 0.005) and trait anxiety (*F*(2, 57) = 21.54, *p* < 0.001). Post-hoc Newman-Keuls test showed that for state anxiety LA (*M* = 33.84, *SD* = 6.29) had significantly lower levels of anxiety compared to MA (*M* = 40.22, *SD* = 7.32; *p* = 0.009) and HA (*M* = 39.79, *SD* = 6.08; *p* = 0.006), while for trait anxiety all groups differed significantly from each other with LA (*M* = 36.42, *SD* = 6.71) showing lower levels of trait anxiety compared to MA (*M* = 44.32, *SD* = 8.05; *p* = 0.001) and HA (*M* = 51.53, *SD* = 7.01; *p* < 0.001) and MA showing lower levels of trait anxiety compared to HA (*p* = 0.003). Correlations between levels of anxiety and differential SCR response were explored to exclude an effect of anxiety on results.

The study was designed and conducted in accordance with the ethical principles of the World Medical Association Declaration of Helsinki and the institutional guidelines of the University of Bologna and was approved by the Ethics Committee of the Department of Psychology. All participants gave informed written consent to participation after being informed about the procedure of the study.

### Stimuli

The task consisted in a classical differential fear conditioning paradigm with partial reinforcement^33^,^34^. Two isoluminant coloured squares represented the CS. The UCS consisted of a mild electric stimluation of 200 ms in duration generated by a Digitimer Stimulator (Model DS7, Digitimer Ltd., UK) administered to the inner wrist of the right hand, to which two electrodes were attached. The intensity of the stimulation was set with a standard workup procedure. It was initially set at 0.5 mA and increased of 1 mA until participants reported it as being highly uncomfortable but not painful.

Each trial consisted in the presentation of one CS in the centre of a computer screen (17″, refresh rate 60 Hz) for 6 seconds followed by an inter trial interval of 12 seconds during which a fixation cross was presented. The task included 40 trials (20 for each CS) divided in three blocks: habituation, acquisition and extinction. At the beginning of habituation, instructions appeared on the screen stating that two different images would be presented one at the time in the centre of the screen, no stimulation would be administered and the task of the participant would be to carefully observe the images. Habituation included 4 trials (2 for each CS) to ensure the absence of any baseline differences within and between groups in response to the images. At the beginning of acquisition similar instructions stated that the same two images would appear one at the time in the centre of the screen and that one of them might be paired with the stimulation. The task of participants remained to carefully observe the images. No information was given about contingencies between images and stimulation. Acquisition included 16 trials (8 for each CS). CS+ was reinforced in 80% of the trials (*n* = 6), while CS− was never reinforced. Extinction followed acquisition without any instructions. It included 20 trials (10 for each CS) and no stimulation was administered. Stimuli were presented in pseudorandomised order, no more than two presentations of the same stimulus occurred in a row^34^. The first two trials of acquisition always included one CS− and one CS+ presented randomly. The colour of the square associated to the CS+ and CS− was counterbalanced across participants.

### Skin conductance response recording

The SCR was recorded through two Ag/AgCl electrodes (TSD203 Model; Biopac Systems, USA), filled with isotonic hyposaturated conductant attached to the distal phalanges of the second and third finger of participants’ left hand and held with Velcro straps. The SCR signal was continuously recorded at 200 Hz and amplified using a DC amplifier (Biopac GSR100; Biopac Systems, USA) with 5 μS/V gain factor and 10 Hz low pass filter. The analogue signal was digitalized using the MP-150 digital converter (Biopac Systems, USA) and fed into AcqKnowledge 3.9 software (Biopac Systems, USA).

### Assessment of subjective anxiety, fear and contingency awareness

At the end of the task, participants were asked to report the level of anxiety and fear experienced at the presentation of each CS during the experiment on separate visual analogue scales ranging from 0 (not at all) to 100 (extreme). The order of questions was balanced across participants.

Participants were also asked to indicate which of the two stimuli was associated with the stimulation to ensure explicit awareness of pairing between CS+ and UCS. Participants who failed to report the correct association were removed from analysis.

### Procedure

The experiment took place in a sound attenuated room with dimmed light. Participants were seated in a chair in front of a computer monitor at ~70 cm distance. Once seated, the experimental procedure was explained and written informed consent was obtained from participants. Then SCR electrodes were attached and correct recording of the signal was ensured. Afterwards, the intensity of the stimulation was set and the task began. Following completion of the task, subjective reports were completed.

### Data analysis

SCR data were analyzed using MATLAB (The MathWorks, Inc., USA) custom-made scripts^33^. SCR was calculated as the peak-to-peak amplitude difference of the largest deflection in the 0.5–4.5 sec latency window after stimulus onset. Regarding SCR to UCS, stimulus onset was represented by the time of stimulation administration, while regarding SCR to CS, stimulus onset referred to the time of CS appearance. The SCR was transformed into microsiemens (μS) and calculated for each trial. Minimum response criterion was 0.02 μS and smaller responses were encoded as zero. Square root transformation was conducted on raw SCR to normalize the data distribution and SCR were scaled to each subject’s maximal UCS response to account for interindividual variability^34^.

Both SCR to UCS and CS− were analysed to ensure that groups did not differ in their physiological response to the stimulation or in the anticipatory response to CS− but only to a conditioned stimulus that predicts an aversive event (i.e. CS+). Regarding SCR to UCS, both peak response and average response were analysed. For each participant, peak response represented the highest SCR in response to the six stimulations administered while mean response was the average of the SCRs to the six stimulations. Regarding the response to CSs, SCRs during the three phases of conditioning were analysed separately. Concerning habituation, all trials were included in the analysis. With regards to acquisition, the first two trials were not included in the analysis because participants learned the association between UCS and CS+ after its first pairing with the stimulation. Regarding extinction, all trials were included in the analysis but this phase was divided in two blocks (early and late extinction). Then, mean SCR of each participant was computed to produce four average scores representing the SCR of each subject during habituation, acquisition, early and late extinction. These were then averaged to obtain the SCR during the different phases for each alexithymia group.

Assumptions of normal distribution were verified. Several ANOVAs were then used to investigate differences among the three groups. Post hoc analyses were conducted with Newman-Keuls test. Significance threshold was *p* < 0.05.

## Additional Information

**How to cite this article**: Starita, F. *et al.* Reduced anticipation of negative emotional events in alexithymia. *Sci. Rep.* **6**, 27664; doi: 10.1038/srep27664 (2016).
